# Tailored co-localization analysis of intracellular microbes and punctum-distributed phagosome–lysosome pathway proteins using ImageJ plugin EzColocalization

**DOI:** 10.1038/s41598-020-79425-5

**Published:** 2021-01-13

**Authors:** Kang Wu, Bo Yan, Douglas B. Lowrie, Tao Li, Xiao-Yong Fan

**Affiliations:** 1 Shanghai Public Health Clinical Center, Key Laboratory of Medical Molecular Virology of MOE/MOH, Fudan University, Shanghai, 201508 China; 2 TB Center, Shanghai Emerging and Re-emerging Institute, Shanghai, 201508 China

**Keywords:** Cellular microbiology, Data processing

## Abstract

Immunofluorescence is indispensable to monitor redistribution of proteins involved in phagosome–lysosome association pathway-relevant (P–LApr) proteins. The software digitizing the signals of these proteins in an unbiased and automated manner is generally costly and not widely available. The open-source ImageJ plugin EzColocalization, which is for co-localization analysis of reporters in cells, was not straightforward and sufficient for such analysis. We describe here the input of custom Java code in a novel tailored protocol using EzColocalization to digitize the signals of punctum-distributed P–LApr proteins co-localized with phagosomes and to calculate percentages of phagosomes engaged. We showed that SYBR Gold nucleic acid dye could visualize intracellular mycobacteria that did not express a fluorescent protein. This protocol was validated by showing that IFN-γ enhanced the co-localization of a punctum-distributed P–LApr protein (LC3) with *Mycobacterium bovis* BCG in the monocyte/macrophage-like RAW264.7 cells and that there was greater co-localization of LC3 with BCG than with *M. tuberculosis* H37Rv in bone marrow-derived macrophages (BMDMs). Although BCG and a derived strain (rBCG-PA) showed a similarly high degree co-localization with LC3 in BMDMs, in RAW264.7 cells BCG showed much less co-localization with LC3 than rBCG-PA indicating the need for caution in interpreting biological significance from studies in cell lines.

## Introduction

Host cell phagosome–lysosome association (P–LA) pathways become activated in response to cellular engulfment of microbes. The P–LA pathways are an integral feature of innate immunity whereby microbes can be destroyed by the acidic and degradative lysosomes. Subsequently, the antigens from the degraded microbes can be presented to lymphocytes to prime the adaptive host immune response^1^. Many pathogenic intracellular microbes have evolved ways to inhibit and/or escape the P–LA pathways^1^.

Deciphering the signals underlying the responsiveness of a P–LA pathway is generally done by inspection of single cells, e.g. by immunofluorescence^2^, flow cytometry^3^, single-cell sequencing^4^ and by approaches based on analysis of extracts from the bulk of cells, e.g. by Western-blotting^5^, transcriptomics^3^. The immunofluorescence-based analysis of co-localization is indispensable since it can provide confocal images as intuitive evidence of phagosome–lysosome association by rendering visible the distribution of both the pathway-relevant (P–LApr) proteins and the microbes. As a prelude to statistical analysis of the changes in distribution, the visual signals from the host cell P–LApr proteins must be automatically digitized for unbiased statistical tests. This can present a challenge for the average researcher. There are specifically designed equipment/microscopes and accompanying imaging software that can achieve the digitization task (e.g. Nikon Eclipse TiE/B automated fluorescent microscope with NIH-Elements DUO software^2,6^, or BioTek Gen5 Image Prime). However, many laboratories do not have access to such high-cost tools. Open-source standalone software that can achieve the sophisticated analysis of output from alternative fluorescence microscopes is needed.

The open-source ImageJ plugin EzColocalization was created for the co-localization analysis of two or three reporters in cells^7^. EzColcalization also allows researchers to input custom Java code, and then to output parameters of interest^7^. The software is not straightforward and sufficient to be used for digitizing the signal values of the P–LApr proteins co-localized with microbes or for calculating the relative degrees P–LApr co-localization with different microbes. Here we outline the tailored use of EzColocalization with customized Java code for digitizing the signals of punctum-distributed P–LApr proteins co-localized with intracellular microbes in phagosomes. Our subjects for analysis were murine macrophage cell line RAW264.7 and bone marrow-derived macrophages (BMDMs) with intracellular *Mycobacterium bovis* bacille Calmette-Guérin (BCG) and *Mycobacterium tuberculosis* H37Rv (*Mtb*) as examples. LC3 is associated with P–LA pathways post the engulfment of mycobacteria by phagocytes^2^. LC3 typically displays a punctum-like distribution pattern within cells, especially after mycobacterial infection. This is also often the case for other P–LApr proteins (e.g. LAMP1, ATG5, ATG12, p40^phox^, p47^phox^, transferrin, CD63)^2,8–11^, perhaps due to their preferred association with cellular vesicles such as P–LA vesicles. We chose LC3 as a representative punctum-distributed P–LApr protein for immunofluorescent analysis. In developing this method we additionally showed that intracellular mycobacterial strains that do not express a fluorescent protein could be stained in situ and visualized using SYBR Gold nucleic acid dye (SG dye) for subsequent immunofluorescent analysis. This tailored use of EzColocalization software should be applicable to the analysis of other intracellular microbes also.

## Results

### Visualization of intracellular mycobacteria via SG dye and preparation of the “Cell identification input” for the ImageJ plugin EzColocalization

**Figure 1:**
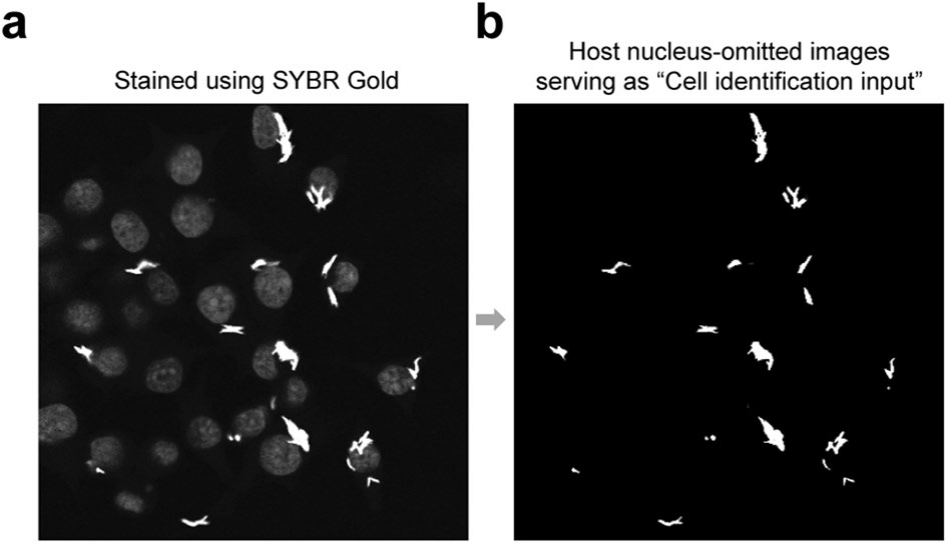
Staining by SG dye of RAW264.7 cells infected with *M. bovis* BCG (**a**), and the omission of stained host cell nuclei (**b**). Host cell nuclei were empirically omitted in ImageJ following the procedure: Image > Adjust > Brightness/Contrast… > Set > type proper ‘minimum displayed value’ (150 for **b**) > Apply.

As shown in Fig. 1a, SG dye simultaneously stained both the host cell nuclei and intracellular BCG strains. However, the stained host cell nuclei and intracellular BCG strains displayed distinctly different signals, which facilitated the empirical omission of host cell nuclei from analysis (Fig. 1b, cutoff pixel value (PV) of 150 for the image). Images such as those shown in Fig. 1b were used as “Cell identification input” for the ImageJ plugin EcColocalization. The software was then used to identify the regions of intracellular bacteria and the corresponding regions of overlap with other monochromatic images (e.g. images of LC3 in this study) to permit extraction of co-localized PVs (e.g. extraction of the PVs of LC3 in this study).

### Protocol to export the mean, maximum, median PVs of punctum-distributed P–LApr proteins co-localized with microbes

**Figure 2:**
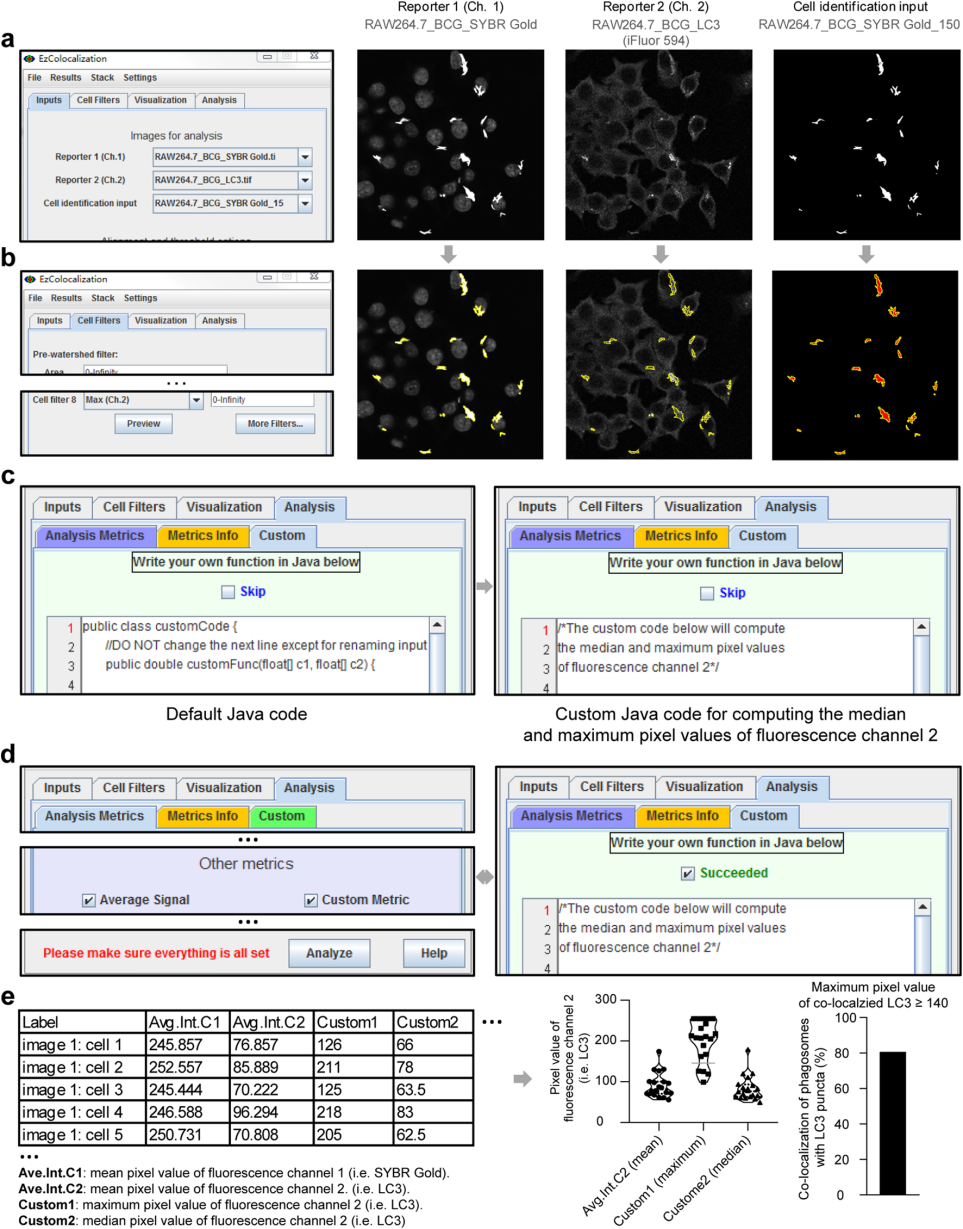
Protocol to export mean, maximum, median pixel values (PVs) of a reporter. (**a**) Inputting the monochromatic images. (**b**) Locating the region of intracellular BCG/phagosomes in “Cell identification input” (yellow margins filled with red), and the corresponding overlapping regions in reporter 1 and reporter 2. (**c**) Inputting custom Java code for exporting maximum and median PVs of reporter 2 in fluorescence channel 2. (**d**, **e**) Exporting and plotting the mean, maximum, median PVs of a reporter, and empirically calculating the percentage of engulfed BCG/phagosomes co-localized with reporter 2 (i.e. LC3 puncta).

A protocol example, used to analyze the images shown in Supplementary Figs. S2–S4, is illustrated in Fig. 2 and described in detail below.

Step 1. In EzColocalization, input monochromatic reporter images of different colors to “Reporter 1(Ch. 1)”, “Reporter 2(Ch. 2)”, and “Cell identification input” of “Inputs” tab via “File” > “Open”. As shown in Fig. 2a, we input Supplementary Fig. S1 “RAW264.7_BCG_SYBR Gold.tif” into “Reporter 1(Ch. 1)”, Supplementary Fig. S2 “RAW264.7_BCG_LC3.tif” into “Reporter 2(Ch. 2)”, and Supplementary Fig. S3 “RAW264.7_BCG_SYBR Gold_150.tif” into “Cell identification input”.

Step 2. Press the button “Preview” of “Cell Filters” tab for selecting intracellular microbes or phagosomes and the corresponding overlapping regions in other monochromatic images. As shown in Fig. 2b, intracellular BCG strains or phagosomes in “Cell identification input” were highlighted with yellow margins filled with red, and then the corresponding regions in “Reporter 1 (Ch. 1)” and “Reporter 2 (Ch. 2)” were also highlighted. Intracellular BCG strains or phagosomes could also be similarly located using another software (i.e. Gen5 Image Prime) (Supplementary Fig. S5).

Step 3, replace the default Java code with the custom Java code in “Custom” sub-tab of the “Analysis” tab for customized computing of the maximum and median PVs of a reporter. As shown in Fig. 2c, the maximum and median PVs of reporter 2 (i.e. LC3 in this study) were computed. See the “Supplementary Method” for the custom Java code.

Step 4. To export the mean, maximum, median PVs of highlighted regions of a reporter, check “Average Signal” and “Custom Metric”, and press the “Analyze” button of the “Analysis Metrics” sub-tab of the “Analysis” tab (Fig. 2d). In accordance. “☑Succeeded” is displayed in the “Custom” sub-tab of the “Analysis” tab (Fig. 2d).

Step 5. Export the file containing mean, maximum, median PVs of a reporter. As shown with this example in the left panel of Fig. 2e, the default mean PVs of reporter 1 (i.e. “Avg.Int.C1”, intracellular BCG/SG dye) and reporter 2 (i.e. “Avg.Int.C2”, LC3) were exported; maximum (i.e. “Custom1”) and median (i.e. “Custom2”) PVs of LC3 were also exported. A plot of the PVs is shown in Fig. 2e middle panel.

The PVs of LC3 puncta were higher than the PVs of basally and evenly distributed cytosolic LC3. Accordingly, an empirically/visually-determined cutoff PV, above which only LC3 puncta were representatively preserved while the signals of basally and evenly distributed cytosolic LC3 were omitted (Supplementary Fig. S4), could be obtained via a similar procedure as in Fig. 1. Then the percentage of BCG/phagosomes that were co-localized with LC3 puncta (i.e. the percentage of phagosomes with maximum LC3 PVs larger than the empirically/visually-determined cutoff PV) was calculated (Fig. 2e middle and right panel).

### Higher co-localization of LC3 with *M. bovis* BCG strains in RAW264.7 cells treated with IFN-γ

**Figure 3:**
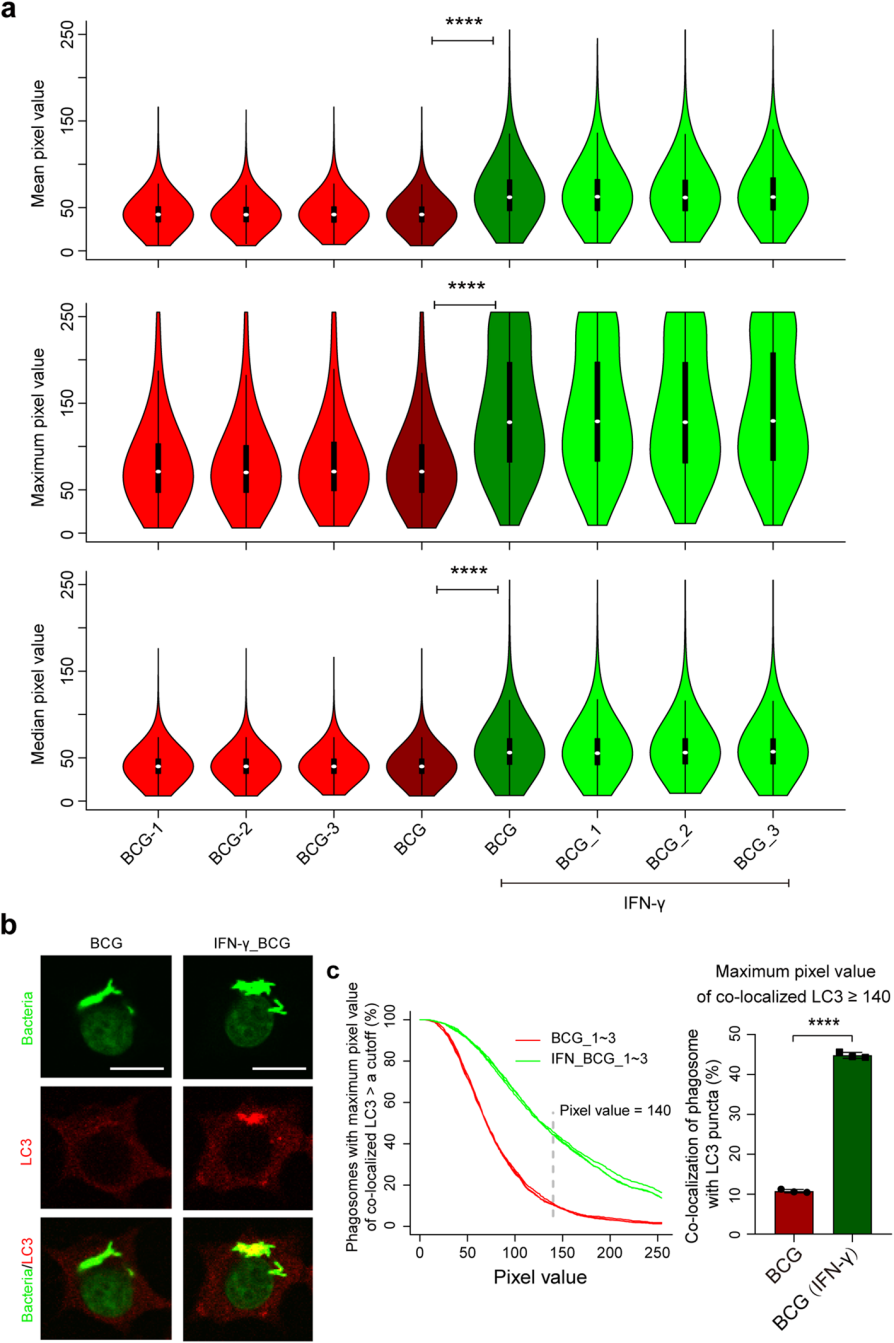
Effect of IFN-γ on co-localization of LC3 with intracellular *M. bovis* BCG strains/phagosomes in RAW264.7 cells. (**a**) Violin plots of mean (upper panel), maximum (middle panel), or median (lower panel) PVs of LC3 co-localized with intracellular BCG/phagosomes. BCG_1–3 shows an individual violin plot of PVs from three coverslips from separate experiments; BCG shows a violin plot of combined PVs of BCG_1–3. There were at least 300 phagosomes/PVs for each violin plot. (**b**) Representative images depicting the co-localization with or without IFN-γ treatment. Scale bars represent 10 μM. (**c**) Cumulative percentages of intracellular BCG/phagosomes that had a corresponding maximum PV of LC3 larger than a cutoff (left panel) or ≥ 140 (right panel). ****P ≤ 0.0001; one-way ANOVA with Tukey’s multiple comparisons test for (**a**), or unpaired and two-tailed Student’s t test for (**c**) right panel.

As shown in Fig. 3, there were significantly higher mean, maximum, median PVs of LC3 co-localized with intracellular BCG/phagosomes in RAW264.7 cells that had been treated with IFN-γ, compared to un-treated RAW264.7 cells. The differences in LC3 co-localization were more visually impressive when shown as violin plots using the custom-exported maximum PVs of LC3, than in plots using the mean and median PVs (Fig. 3a). Based on an empirically-determined cutoff PV of 140, approximately 44.8% of the BCG strains or phagosomes co-localized with LC3 puncta in RAW264.7 cells treated with IFN-γ, whereas only 10.8% co-localized with LC3 puncta in RAW264.7 cells without IFN-γ treatment (Fig. 3c). The same conclusion was reached when digitizing the mean PVs using Gen5 Image Prime, which gave results (Supplementary Fig. S5) similar to those shown in the upper panel of Fig. 3a.

### Higher co-localization with LC3 of BCG compared to H37Rv seen in BMDMs

**Figure 4:**
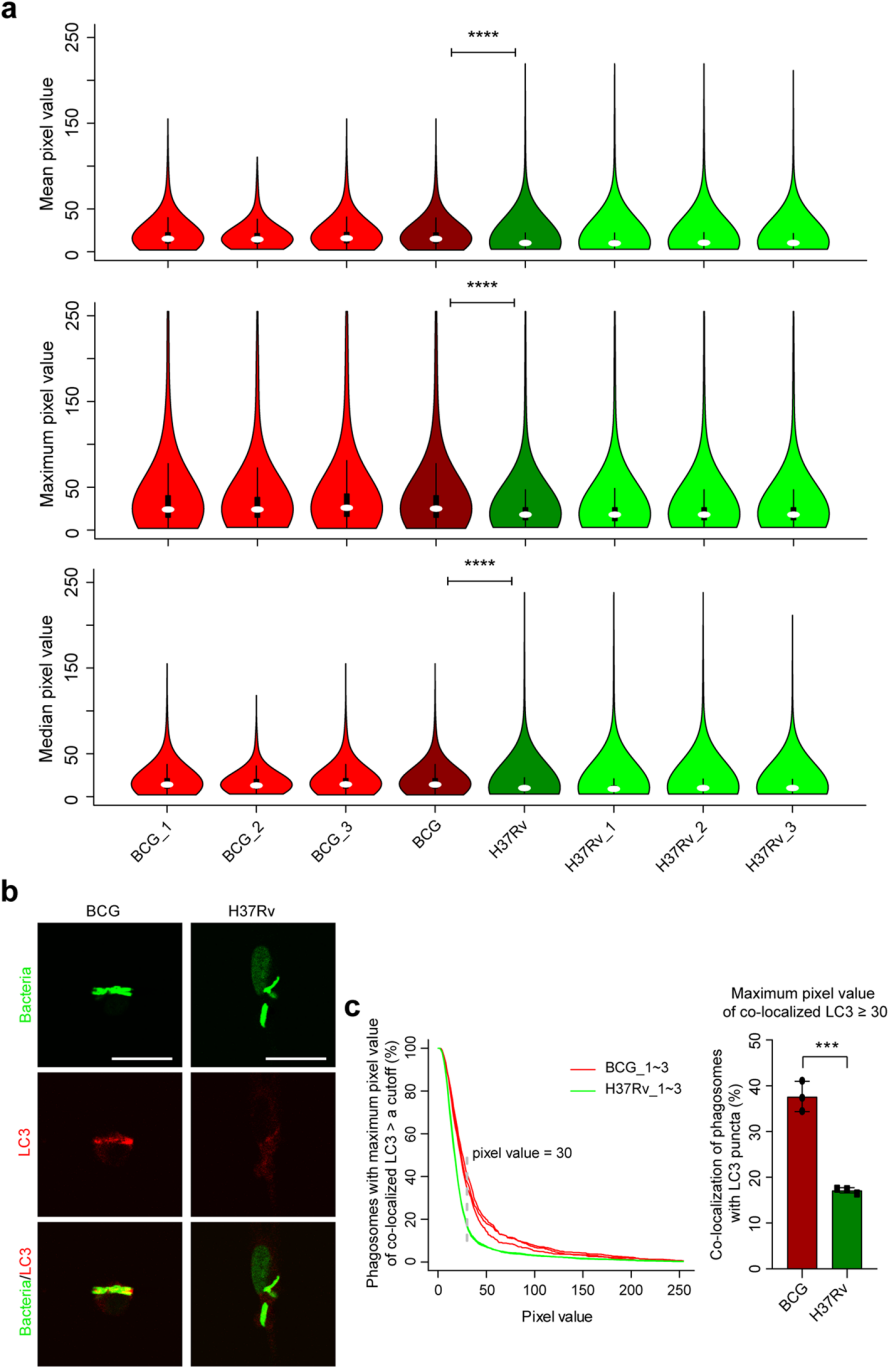
Co-localization of LC3 with intracellular *M. bovis* BCG or *Mtb* H37Rv in BMDMs. (**a**) Violin plots of mean (upper panel), maximum (middle panel), or median (lower panel) PVs of LC3 co-localized with intracellular mycobacteria/phagosomes. BCG_1–3: individual violin plot of PVs from three coverslips from separate experiments; BCG: violin plot of combined PVs BCG_1–3. H37Rv_1–3 and H37Rv indicates corresponding data obtained with H37Rv. There were at least 300 phagosomes/PVs for each violin plot. (**b**) Representative images depicting the co-localization of intracellular mycobacteria/phagosomes with LC3 in BMDMs. Scale bars represent 10 μM. (**c**) Cumulative percentages of bacteria/phagosomes with a corresponding maximum PV of LC3 larger than a cutoff value (left panel) or ≥ 30 (right panel). ***P ≤ 0.001; ****P ≤ 0.0001; one-way ANOVA with Tukey’s multiple comparisons test for (**a**), or unpaired and two-tailed Student’s t test for (**c**) right panel.

BCG, the only licensed live tuberculosis (TB) vaccine, is less virulent than wild-type *M. bovis* and *Mtb*^12–14^. In BMDMs of C57BL/6 mice there were significantly higher mean, maximum, median PVs of LC3 co-localized with intracellular BCG strains/phagosomes, compared to *Mtb* H37Rv (Fig. 4), This was consistent with their different virulence phenotypes. Based on the empirically-determined cutoff PV of 30, approximately 37.7% of intracellular BCG/phagosomes co-localized with LC3 puncta, whereas only 17.1% of H37Rv/phagosomes were so co-localized (Fig. 4c). A similar pattern to that shown in Fig. 4 was also observed in BMDMs of BALB/c mice (data not shown).

### Divergence of LC3 co-localizations with BCG and derived strain expressing PPE17_1–177_–ActA_27–612_ (rBCG-PA) in BMDMs and RAW264.7 cells

The enhancement of LC3 association with BCG/phagosomes by IFN-γ treatment in RAW264.7 cells was readily reproducible; likewise, the differences in LC3 co-localization between intracellular BCG and H37Rv in BMDMs (Figs. 3 and 4) were robust. These findings provided a firm basis for testing for differences between BCG and recombinant BCG strains in these cells; in this example our interest was in comparing BCG and rBCG-PA (Supplementary Fig. S6).

**Figure 5:**
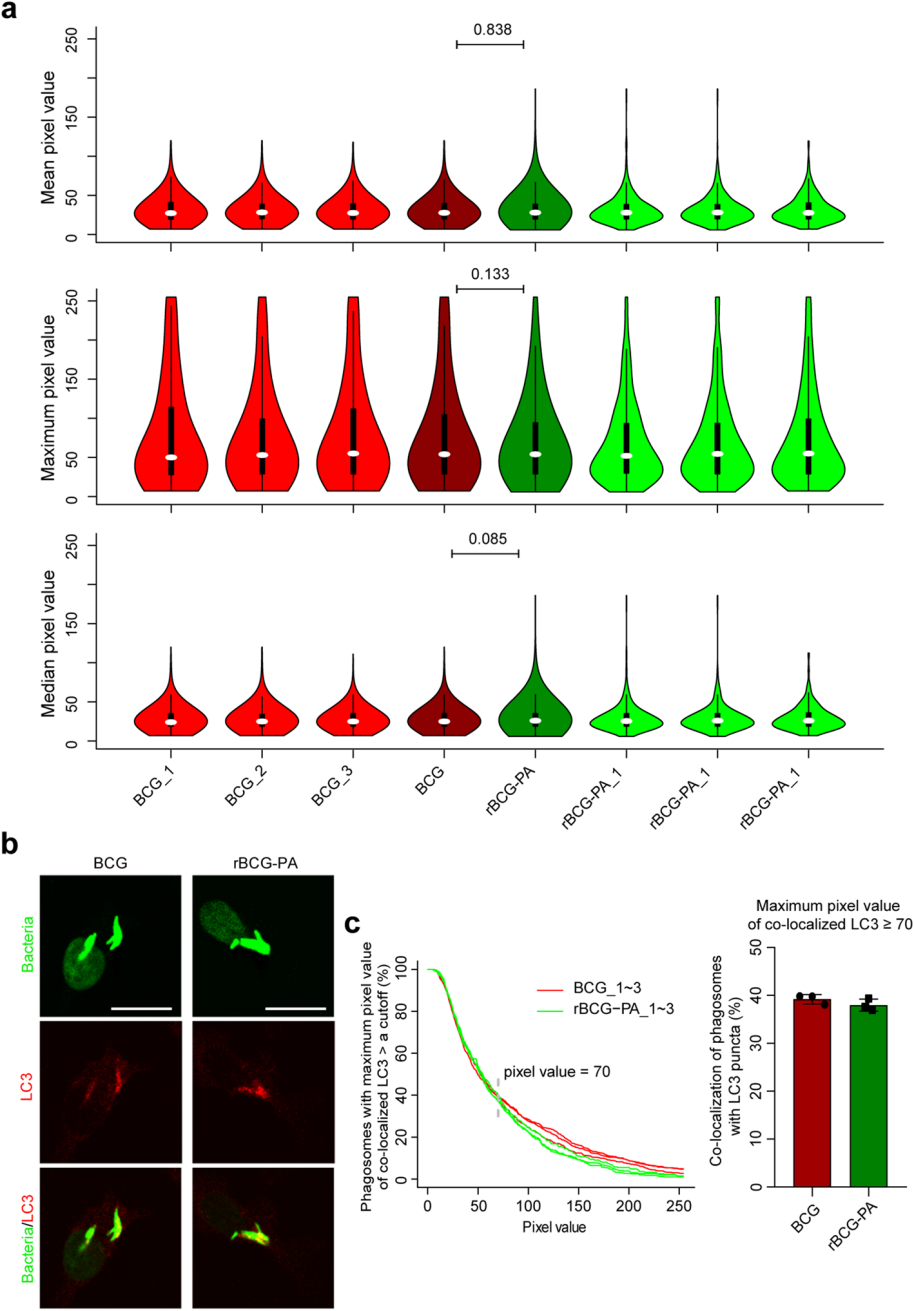
Co-localization of BCG and rBCG-PA strains with LC3 in BMDMs. (**a**) Violin plots of mean (upper panel), maximum (middle panel), or median (lower panel) of LC3 co-localized with phagosomes. BCG_1–3, BCG, rBCG-PA_1–3, and rBCG-PA mean the same as in Fig. 4a. There were at least 200 phagosomes/PVs for each violin plot. (**b**) Representative images depicting the co-localization of BCG and rBCG-PA with LC3 in BMDMs. Scale bars represent 10 μM. (**C**) Cumulative percentages of BCG and rBCG-PA (i.e. phagosomes) that had a corresponding maximum PV of LC3 larger than a cutoff value (left panel) or larger than 70 (right panel). *P* values are marked for differences between BCG and rBCG-PA; one-way ANOVA with Tukey’s multiple comparisons test for (**a**).

**Figure 6:**
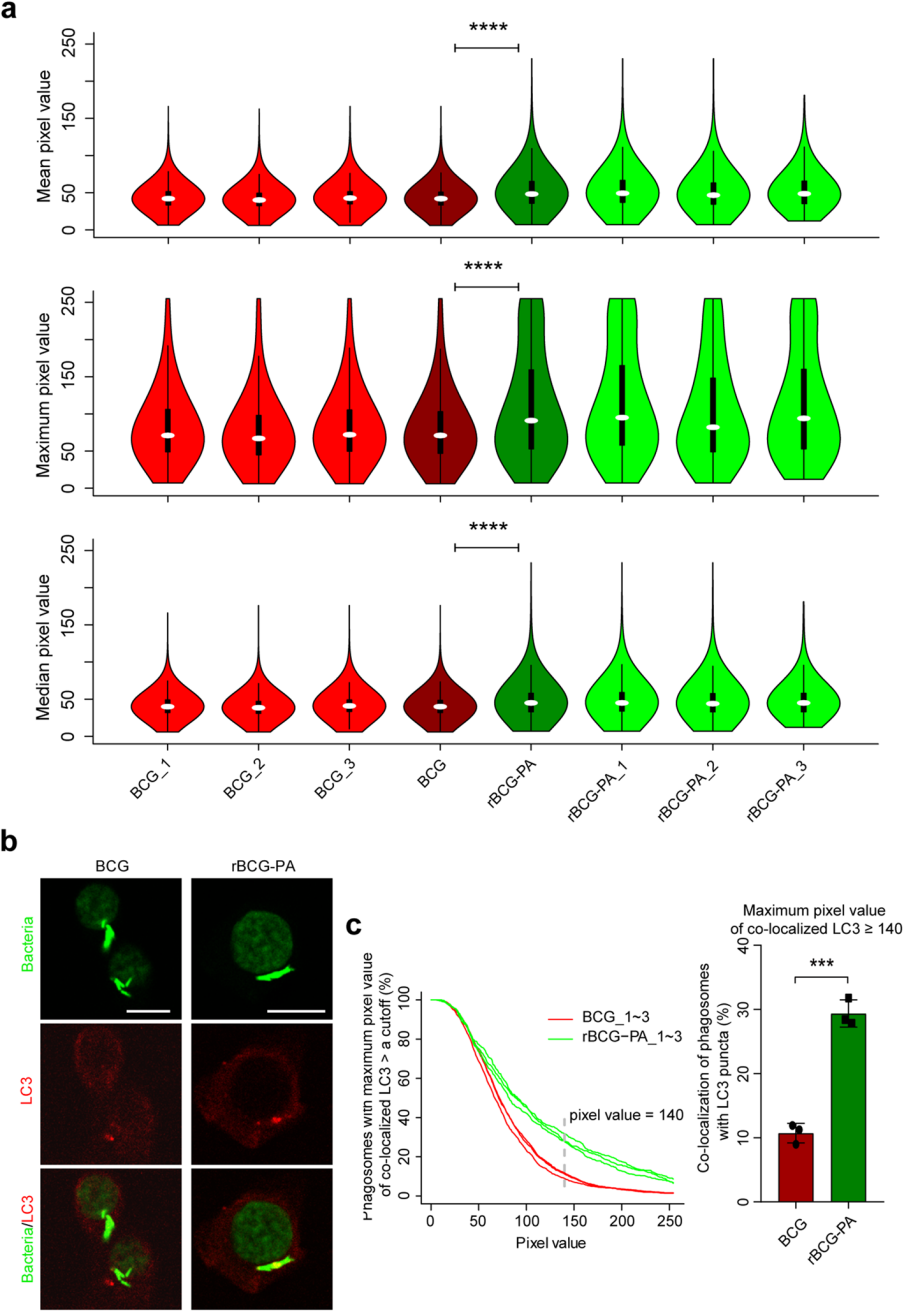
Greater co-localization of rBCG-PA with LC3 compared to BCG in RAW264.7 cells. (**a**) Violin plots of mean (upper panel), median (middle panel), or maximum (lower panel) of LC3 co-localized with phagosomes. BCG_1–3, BCG, rBCG-PA_1–3, and rBCG-PA mean the same as in Fig. 4a. There were at least 200 phagosomes/PVs for each violin plot. (**b**) Representative images depicting the co-localization of BCG or rBCG-PA with LC3 in RAW264.7 cells. Scale bars represent 10 μM. (**c**) Cumulative percentages of intracellular BCG or rBCG-PA (i.e. phagosomes) with a corresponding maximum PV of LC3 larger than a cutoff (left panel) or larger than 140 (right panel). ***P ≤ 0.001; ****P ≤ 0.0001; one-way ANOVA with Tukey’s multiple comparisons test for (**a**), or unpaired and two-tailed Student’s t test for (**c**) right panel.

As shown in Fig. 5, there were no statistically significant differences in mean, maximum, median PVs of LC3 co-localized with either of the intracellular strains/phagosomes in BMDMs. Based on the empirically-determined cutoff PV (i.e. 70), approximately 39% of either strain co-localized with LC3 puncta (Fig. 5c). Contrary to the results in BMDMs, in RAW264.7 cells there were significantly higher mean, maximum, median PVs of LC3 co-localized with intracellular rBCG-PA compared to intracellular BCG (Fig. 6). Based on the empirically-determined cutoff PV (i.e. 140), roughly 10.7% of intracellular BCG co-localized with LC3 puncta, whereas 29.3% of intracellular rBCG-PA strain co-localized with LC3 puncta (Fig. 6c).

## Discussion

Visually assessing the fate of microbes (e.g. *Mtb*) in infected macrophages can provide an invaluable aid to understanding in the process of developing new immunological or chemotherapeutic interventions to overcome the pandemic. Intracellular interactions between the engulfed microbes in phagosomes and the antimicrobial systems of the host cell such as lysosomes are known to be critical for the outcome and are now accessible for inspection by fluorescence microscopy. However, statistical analysis of these interactions and of the distribution of key components of their regulatory pathways can be challenging. In this study, we have demonstrated the use of a refinement of open-source software to facilitate the process.

This investigation proved the feasibility of a tailored strategy/protocol for analyzing the co-localization between punctum-distributed P–LApr proteins and intracellular microbes/phagosomes. Our use of the ImageJ plugin EzColocalization with customized PVs of punctum-distributed P–LApr proteins was probably the first endeavor to calculate the relative percentages of such co-localizations using the maximum PVs of P–LApr proteins. Additionally, it provided the first exemplified test of visualizing intracellular mycobacteria strains with SG dye for immunofluorescence analysis.

Compared to other applications (e.g. JACoP^15^, Coloc2^16^, Wright Cell Imaging Facility^17^, MatCol^18^), one of the main features of EzColocalization was its easy-to-use built-in option for selecting many objects in an image, which thus facilitated the targeted selection of intracellular bacteria/phagosomes. We adopted the pragmatic assumption that all intracellular bacteria were in phagosomes. Images prepared for “Cell identification input” were used to select intracellular bacteria/phagosomes (Fig. 2a). It was easy to perceive that, for a strain expressing a fluorescent protein (e.g. green fluorescent protein, GFP), monochromatic image capturing this fluorescent channel could be used for “Cell identification input”.

EzColocalization enabled analysis based on two or three reporter channels and end users could export custom maximum or median PVs of fluorescence channels 1, 2, or 3 at their desire. Although throughout this study we mainly demonstrated the analysis based on two reporter channels, custom Java codes for analysis based on both two and three reporter channels are provided (see “Supplementary File” and Supplementary Fig. S7), and the results from using two or three channels were essentially identical (Supplementary Table S1).

Mean (default)/maximum (custom)/median (custom) PVs could all be used for plotting and analysis, and all displayed consistent statistical power (Figs. 3, 4, 5, 6). However, due to the more or less unavoidable saturation of some PVs (Figs. 3, 5 and 6, middle panels), mean and maximum PVs were sometimes distorted, whereas the custom median PVs are mathematically objective. However, it was regarded as visually more impressive when plotted using maximum PVs, rather than using mean and median PVs (Figs. 3a and 6a). At any time, when beginning to capture images, parameters should be technically adjusted and fixed to minimize the saturation of PVs.

We also showed that intracellular mycobacteria that do not express a fluorescent protein, could be visualized with SG dye and readily distinguished from the background. The SG dye staining protocol used in this study specifically and intensely stained in vitro cultured mycobacteria, but not various other Gram-positive and Gram-negative bacteria^19^. SG dye was reported to stain 99% of in vitro cultured *Mtb*, irrespective of whether they were actively replicating bacteria or non-replicating hypoxic bacteria and the signal remained unaltered for as long as 21 months when kept at room temperature^19^. Here in our example, SG dye stained the DNA of both the intracellular mycobacteria and the host cell nuclei with a sharp difference of PVs, which made it possible to omit images of host cell nuclei and only preserve those of intracellular mycobacteria (Fig. 1). Then the host cell images with nuclei-omitted could be used as “Cell identification input”.

Crucially, we found that the treatment of the infected cells with the SG staining operation did not affect the detection of LC3. This was also the situation with other widely used P–LApr markers such as LAMP-1, p40^phox^, and gp91^phox^ (data not shown) but we also observed that the SG staining operation completely abolished the intracellular ROS signals that were detected via carboxy-H2DCFDA (data not shown). Thus, the compatibility of the SG method with other markers must be verified. It should be noted that the data underlying each of the Figs. 3, 4, 5 and 6 were obtained under the same conditions (e.g. voltage), however, the images represented in the three figures were not always captured using the same parameters. This was why different cutoffs of PVs of LC3 were empirically/visually determined to omit the basally and evenly distributed cytosolic signal of LC3 and preserve the LC3 puncta (Figs. 3, 4, 5, 6).

BCG, the only licensed live tuberculosis vaccine, is less virulent than wild-type *M. bovis* and *Mtb*^12–14^ and elicits immune activation via cytokines such as IFN-γ that are central to vaccine-derived immunity. Thus, the enhancement of co-localization of LC3 with intracellular BCG by IFN-γ seen here (Fig. 3) was consistent with earlier studies^8^ and provided confirmation of the validity of our SG method. Similarly, the greater association of LC3 with BCG than with H37Rv (Fig. 4) was consistent with the differences in virulence.

The difference between strains of BCG in their propensity to trigger P–LA in some host cells and not others (Figs. 5 and 6) was striking and awaits further exploration. Although monocyte/macrophage-like tumor cell lines such as RAW264.7 cells are useful for many research purposes, they have many altered properties as a result of their transformation. With approximately the same numbers of rBCG-PA bacteria per cell, the P–LA was approximately the same in RAW264.7 cells (29.3%, Fig. 6) as in BMDMs (39%, Fig. 5), suggesting there was no fundamental deficiency in capacity for P–LA in the cell line. Because BCG also elicited about 39% P–LA in BMDMs (Fig. 5) but only about 10.7% in RAW264.7 cells (Fig. 6), although this could be compensated by IFN-γ-treatment (Fig. 3), there may be differences in the P–LA pathways functional in the RAW264.7 cell line. This likewise remains to be explored. The host cells of mycobacteria in vivo are certain to be in micro-environments in foci of infection that differ from those that pertained here, namely the adherence to artificial surfaces in artificial culture media. Analysis of P–LA pathways in vivo will present further challenges.

PPE17_1–177_ (P) is a surface localizing domain and could be utilized to deliver heterologous proteins (e.g. ActA_27–612_ (A) of rBCG-PA in this study) onto the mycobacterial surface^20^. The surface-localized native ActA in *Listeria monocytogenes* (*Lm*) mediates the lower co-localization of the bacteria with LC3, once they move from phagosomes to cytosol^21^. BCG does not become exposed to cytosol. It does not exit from phagosomes due to a genomic deletion of the region of difference 1 (RD1) that harbors genes expressing the proteins (e.g. ESAT-6 and CFP10) that disrupt phagosome integrity^22^. Replacing ActA_27–612_ with irrelevant exogenous proteins (e.g. ovalbumin) in rBCG-PA will be needed to establish if the higher co-localization with LC3 in RAW264.7 cells (Fig. 6) is ActA_27–612_-specific or not. Further experiments might then decipher the mechanisms involved.

In conclusion, we describe a tailored use of the open-source ImageJ plugin EzColocalization for digitizing the PVs of punctum-distributed P–LApr proteins that co-localize with intracellular microbes/phagosomes, and calculating the relative percentages of phagosomes co-localized with the punctum-distributed P–LApr proteins.

## Materials and methods

### Experimental mice and ethics statement

Female specific pathogen-free (SPF) C57BL/6 and Balb/c mice aged 6–8 weeks were purchased from Shanghai Laboratory Animal Research Center (Shanghai, China) and kept under SPF conditions with food and water ad libitum. This study was performed in accordance with the recommendations in the Guide for the Care and Use of Laboratory Animals, and the protocol was approved by the Laboratory Animal Ethical Board of Shanghai Public Health Clinical Center.

### Bacterial strains and growth conditions

*Mycobacterium bovis* BCG Pasteur and its derived rBCG-PA (Supplementary Fig. S6) and *Mtb* H37Rv were grown at 37 °C in liquid Middlebrook 7H9 broth (BD Difco, USA) supplemented with 10% (v/v) oleic acid-albumin-dextrose-catalase enrichment (OADC; BD Difco, USA), 0.5% glycerol and 0.05% Tween-80. Cultures in exponential growth were frozen at − 80 °C after the addition of glycerol (final concentration: 10%). When required, kanamycin was added to a final concentration of 50 μg/mL.

### Cell culture

RAW264.7 cells were grown at 37 °C with 5% CO_2_ in DMEM (Hyclone, China) with 10% heat-inactivated fetal bovine serum (FBS; Biological Industries, USA).

To obtain BMDMs, bone marrow cells (5 × 10^6^) were seeded into 10 mL growth medium (DMEM medium supplemented with 10% (v/v) FBS, 1 × penicillin/streptomycin, and 20 ng/mL r-MCSF). Fresh growth medium (5 mL) was added on day 3. On day 6, BMDMs were used for the experiments.

### Cell infection

RAW264.7 (1 × 10^5^ cells/well/500 μL medium) or BMDMs (2 × 10^5^ cells/well/500 μL medium) were seeded into 24-well plates pre-loaded with round coverslips. One day later, the cells were infected with the mycobacteria strains at a multiplicity of infection (MOI) of 5. Three hours post-infection, extracellular bacteria were removed by triple-washing with 500 μL pre-warmed fresh medium. Finally, 500 μL fresh medium (with or without 200 U/mL IFN-γ; Peprotech, USA) was added and incubated at 37 °C with 5% CO_2_ for 20 h prior to assay.

### Visualization of intracellular mycobacteria strains

The mycobacteria strains used in this study did not express a fluorescent protein, for visualizing the intracellular mycobacteria by subsequent immunofluorescence, the infected cells were stained with SYBR Gold nucleic acid dye (SG dye) (Thermo Fisher Scientific, USA)^19,23^ as follows.

Cells were fixed with 500 μL 4% paraformaldehyde in PBS for 20 min and then rinsed twice with 500 μL PBS. Then the infected cells were permeabilized with 500 μL 0.2% Triton-X100 (dissolved in PBS) for 5 min and then rinsed triple in 500 μL PBS. Cells were stained with 200 μL SG dye (Thermo Fisher Scientific, USA), which had been diluted 1:1000 in stain solution (0.85 M phenol in a 60% glycerol/14% isopropanol solution in distilled water) at 65 °C for 5 min. After cooling the cells at room temperature for an additional 5 min, cells were washed with 500 μL acid alcohol (0.5% HCl in 70% isopropanol) for 3 min^19,23^. To be noted, both the host cell nuclei and the intracellular mycobacteria (i.e. in essence, the nucleic acid of the mycobacteria) were stained by SG dye and displayed green fluorescence under fluorescence microscopy, with intracellular mycobacteria being much brighter than host cell nuclei.

### Immunofluorescence

After fixation, permeabilization, and staining with SG dye, cells were blocked with 500 μL PBS containing 5% skimmed milk overnight at 4 °C. Then cells were incubated with rabbit anti-LC3 antibody (MBL, Japan) at room temperature for 1.5 h, triple-washed with 500 μL PBS, incubated with iFluor 594 goat anti-rabbit IgG (AAT Bioquest, USA) at room temperature for 1.5 h, and finally triple-washed with 500 μL PBS. The coverslips were mounted in Antifade Mounting Medium (Biosharp, China) and examined by confocal microscopy with 40 × objective (microscope model; Leica, Germany). Images were captured from three coverslips in replicated experiments. Tile scanning (i.e. scanning multiple images and joining them into a complete image) was utilized for ensuring the capture of sufficient numbers of intracellular microbes and phagosomes into an image and thereby facilitated image analysis. Monochromatic (8-bit) TIFF images were exported for subsequent analysis using EzColocalization. EzColocalization accepts 8-bit, 16-bit, or 32-bit monochromatic images in a format such as TIFF that retains the original PVs^7^.

### Digitizing the signals of host cell P–LApr proteins and intracellular mycobacteria, and calculating percentage co-localization using the ImageJ plugin EzColocalization

EzColocalization is an open-source plugin of ImageJ^7^. The ImageJ application (version 1.8.0_172) could be found at https://imagej.nih.gov/ij/download.html. The EzColocalization plugin (version named as EzColocalization_.jar-20191007094904) could be found at https://sites.imagej.net/EzColocalization/plugins/. EzColocalization was created for co-localization analysis of two or three reporters in cells. We found that the software was not straightforward and sufficient to be used for digitizing the signals (e.g. maximum and median signals) of P–LApr proteins co-localized with microbes. Neither was it adequate for calculating relative percentages of different microbes co-localized with P–LApr proteins. However, EzColocalization allows the input of custom Java code and subsequent output of a data table containing the custom parameters in addition to the default metrics. We took advantage of (1) the differential green fluorescence intensities between host cell nuclei and intracellular mycobacteria stained with SG dye, and (2) the possibility of output of custom parameters via custom Java code in EzColocalization. Accordingly, we tailored the EzColocalization program and used it to digitize the signals of host cell P–LApr proteins that co-localized with intracellular microbes or phagosomes, and calculated the relative percentage of different microbes co-localized with P–LApr proteins, by using the maximum or median PVs (calculated by the custom Java code) or the mean PVs (default) of P–LApr proteins co-localized with microbes.

As required by EzColocalization^7^, monochromatic images (in the format of TIFF in this study) were exported from the Leica confocal microscope for subsequent analysis. For example, if the cells were stained or captured by two different colored reporters, then two monochromatic images were exported. One monochromatic image for color/reporter one, another monochromatic image for color/reporter two.

### Statistical analysis

The unpaired, two-tailed Student’s t test or one-way ANOVA with Tukey’s multiple comparisons test were used to access the statistical significance of the comparisons of experimental groups using GraphPad Prism software (version 8.0.1) (https://www.graphpad.com/) or R programming language (version 4.0.2) (https://www.r-project.org/). Violin plots were generated using GraphPad Prism software or the package ‘vioplot’ of R software.

## Supplementary Information

Supplementary Information.
