# Supplementary material for: Tailored co-localization analysis of intracellular microbes and punctum-distributed phagosome–lysosome pathway proteins using ImageJ plugin EzColocalization
Source: Sci Rep. 2021 Jan 13;11:1096. doi: 10.1038/s41598-020-79425-5 (PMC7807018; doi:10.1038/s41598-020-79425-5)
Supplement: Supplementary file 1 — Supplementary Information. [file 41598_2020_79425_MOESM1_ESM.pdf]

Tailored co-localization analysis of intracellular microbes and punctum-distributed phagosome-lysosome pathway proteins using ImageJ plugin EzColocalization

Kang Wu[1,2], Bo Yan[1], Douglas B. Lowrie[1,2], Tao Li[1,2*], Xiao-Yong Fan[1,2,*]

[1]*Shanghai Public Health Clinical Center, Key Laboratory of Medical Molecular Virology of MOE/MOH, Fudan University, Shanghai 201508, China*

[2] *TB center, Shanghai Emerging and Re-emerging Institute, Shanghai 201508, China*

[*]Correspondence:

Tao Li, litaokc@126.com ;

Xiao-Yong Fan, xyfan008@fudan.edu.cn.

Running title: A tailored co-localization assay of EzColocalization

Supplementary methods

Custom Java code for 2 reporter channels

```
/*The custom code below will compute
the median and maximum pixel value
of fluorescence channel 2*/

import java.util.ArrayList;
import java.util.Collections;
public class customCode {
	public double[] customFunc(float[] c1, float[] c2) {
		/*c1 and c2 are arrays of pixel values of
		fluorescence channels in the same cell*/
		double maxVal = maximum_value(c2);
		double medianVal = median_value(c2);
		return new double[]{maxVal, medianVal};
	}

	private double maximum_value(float[] arr) {
		float max = arr[0];
		for (int i =1; i<arr.length; ++i) {
			if(max < arr[i]) {
				max = arr[i];
			}
		}
		return max;
	}

	private double median_value(float[] arr) {
		ArrayList<Float> arrList = new ArrayList<Float>();
		for(int i =0; i< arr.length; ++i) {
			arrList.add(arr[i]);
		}
		Collections.sort(arrList);
		if(arrList.size()%2 == 0 ) {
			Float v1 = arrList.get(arrList.size()/2 - 1);
			Float v2 = arrList.get(arrList.size()/2 );
			return (v1+v2)/2;
		} else {
			return arrList.get(arrList.size()/2);
		}
	}
}
```

Custom Java code for 3 reporter channels

```
/*The custom code below will compute
the median and maximum pixel value
of fluorescence channel 2*/

import java.util.ArrayList;
import java.util.Collections;
public class customCode {
	public double[] customFunc(float[] c1, float[] c2, float[] c3) {
		/*c1, c2 and c3 are arrays of pixel values of
	       fluorescence channels in the same cell*/
		double maxVal = maximum_value(c2);
		double medianVal = median_value(c2);
		return new double[]{maxVal, medianVal};
	}

	private double maximum_value(float[] arr) {
		float max = arr[0];
		for (int i =1; i<arr.length; ++i) {
			if(max < arr[i]) {
				max = arr[i];
			}
		}
		return max;
	}

	private double median_value(float[] arr) {
		ArrayList<Float> arrList = new ArrayList<Float>();
		for(int i =0; i< arr.length; ++i) {
			arrList.add(arr[i]);
		}
		Collections.sort(arrList);
		if(arrList.size()%2 == 0 ) {
			Float v1 = arrList.get(arrList.size()/2 - 1);
			Float v2 = arrList.get(arrList.size()/2 );
			return (v1+v2)/2;
		} else {
			return arrList.get(arrList.size()/2);
		}
	}
}
```

Supplementary materials and methods

Preparation of the recombinant BCG strain

$PPE17_{1\text{-}177}$ and *$ActA_{27\text{-}612}$* were cloned from *Mtb* H37Rv and a *Listeria monocytogenes* isolate (courtesy of Hengan Wang of Shanghai Jiaotong University, Shanghai, China) respectively, and sequentially linked in-frame into the multiple cloning site of pMFA42[1,2]. The promoter driving the transcription of PA was a double-mutated furA (Rv1909c) promoter[1,2]. The DNA fragment including both promoter and *$PPE17_{1\text{-}177}$-$ActA_{27\text{-}612}$* (*PA*) was cut from pMFA42 and linked into pMV306 plasmid[3]. The pMV306-based plasmid can site-specifically integrate into the genome of BCG. The resulting plasmid construct was used to electrophorate BCG. The electropharated BCG strains were plated/selected at 37℃ on Middlebrook 7H11 agar containing kanamycin (50 μg/mL). Recombinant BCG clones were grown to exponential phase at 37℃ in 7H9 broth, and verified by Western blotting using antibody against ActA (LSBio, USA). The selected recombinant BCG was named as strain rBCG-PA.

Digitizing the signals of host cell LC3 and intracellular BCG using Gen5 Image Prime

Gen5 Image Prime (version 3.10.06) (https://www.biotek.com/products/accessories/gen5-image-prime/) (BioTec, USA) was also used to digitized the signals of host cell LC3 and intracellular BCG, with the aim of cross validate the performance of EzColocalization. Monochromatic (8-bit) TIFF images (three images as in Fig. 2a) were imported into the software. When importing the images, each image was pseudo-colored differently (e.g. green, red, and gray), and merged together, and changed to 16-bit images in default. Intracellular BCG/phagosomes were located based on the images as described for "Cell identification input" in Fig. 2a. The software could only display and export mean PV of the located regions of each image. To be noted, since the images were changed to 16-bit, so the mean PVs exported were in a range between 0 and 65535 (2^{16}) (Supplementary Fig. S5), rather than in the range between 0 and 255 (2^8) (Fig. 2e).

Supplementary figures

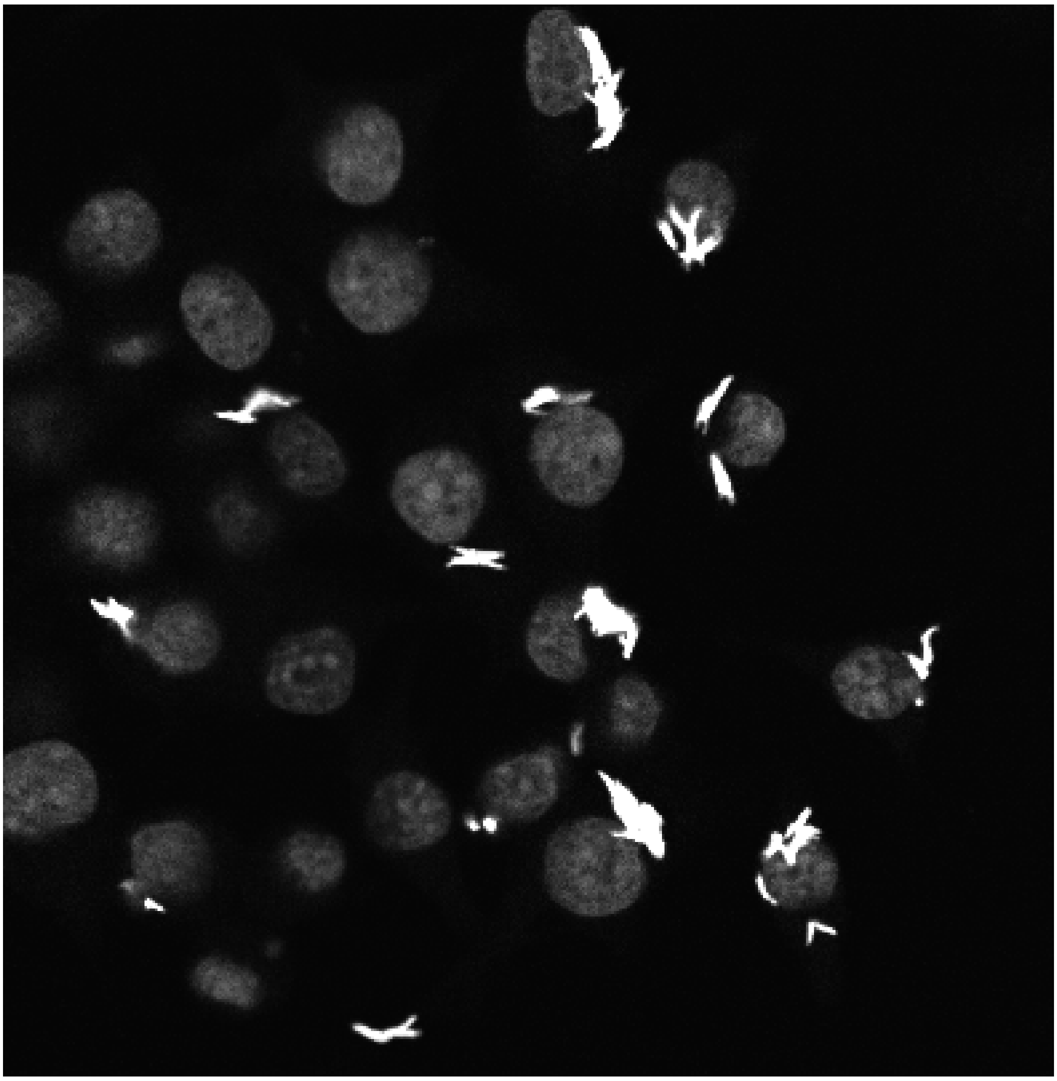

Figure S1. SG dye-stained RAW264.7 cells pre-infected with BCG strains. Three hours post-infection, extracellular strains were removed via triple washing with pre-warmed medium, and incubated in 500 μL fresh medium containing 200 U/mL IFN-γ (Peprotech, USA) at 37℃ with 5% CO_2 for 20 h. Then the cells were used for SG dye staining and immunofluorescence.

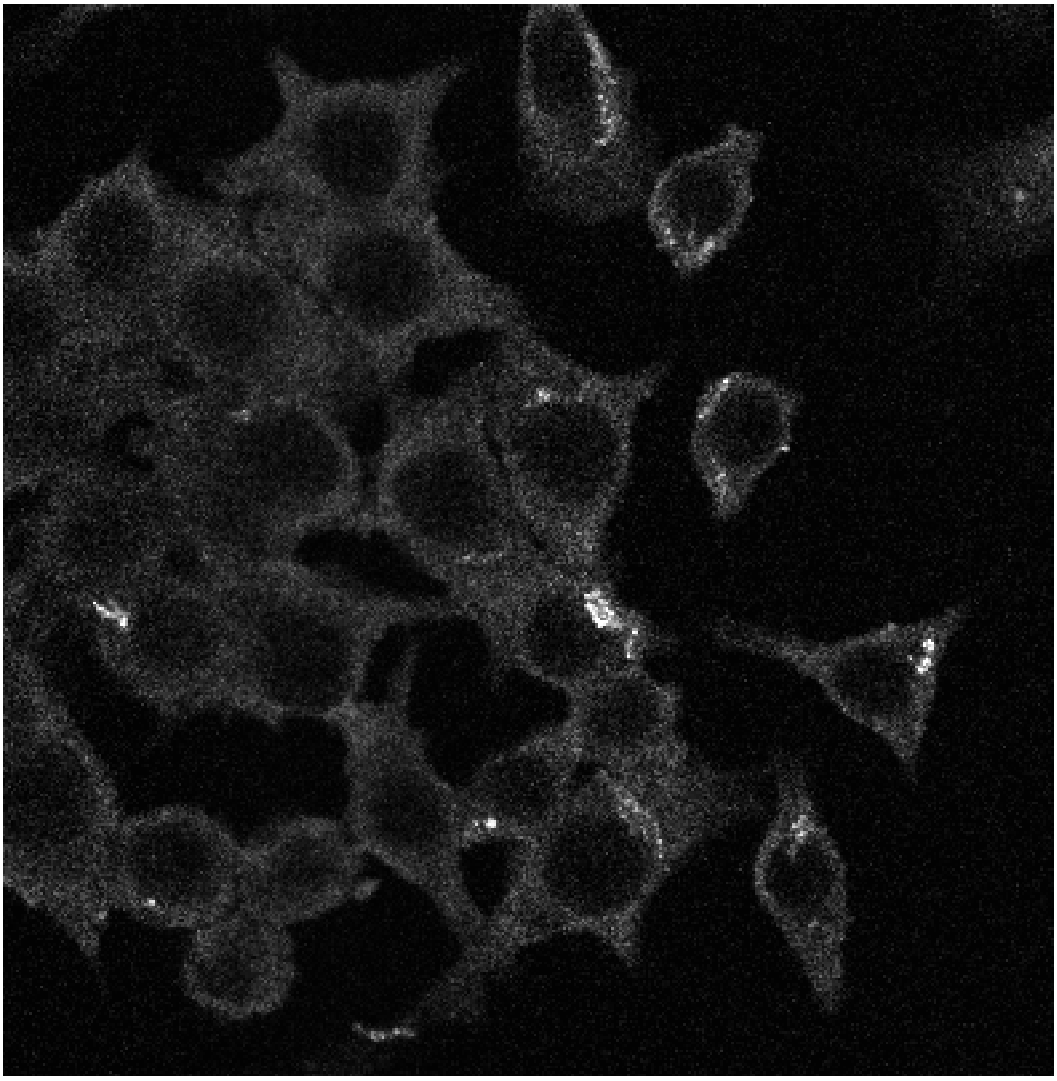

Figure S2. Immunofluorecence of LC3 of cells from Supplementary Fig. S1.

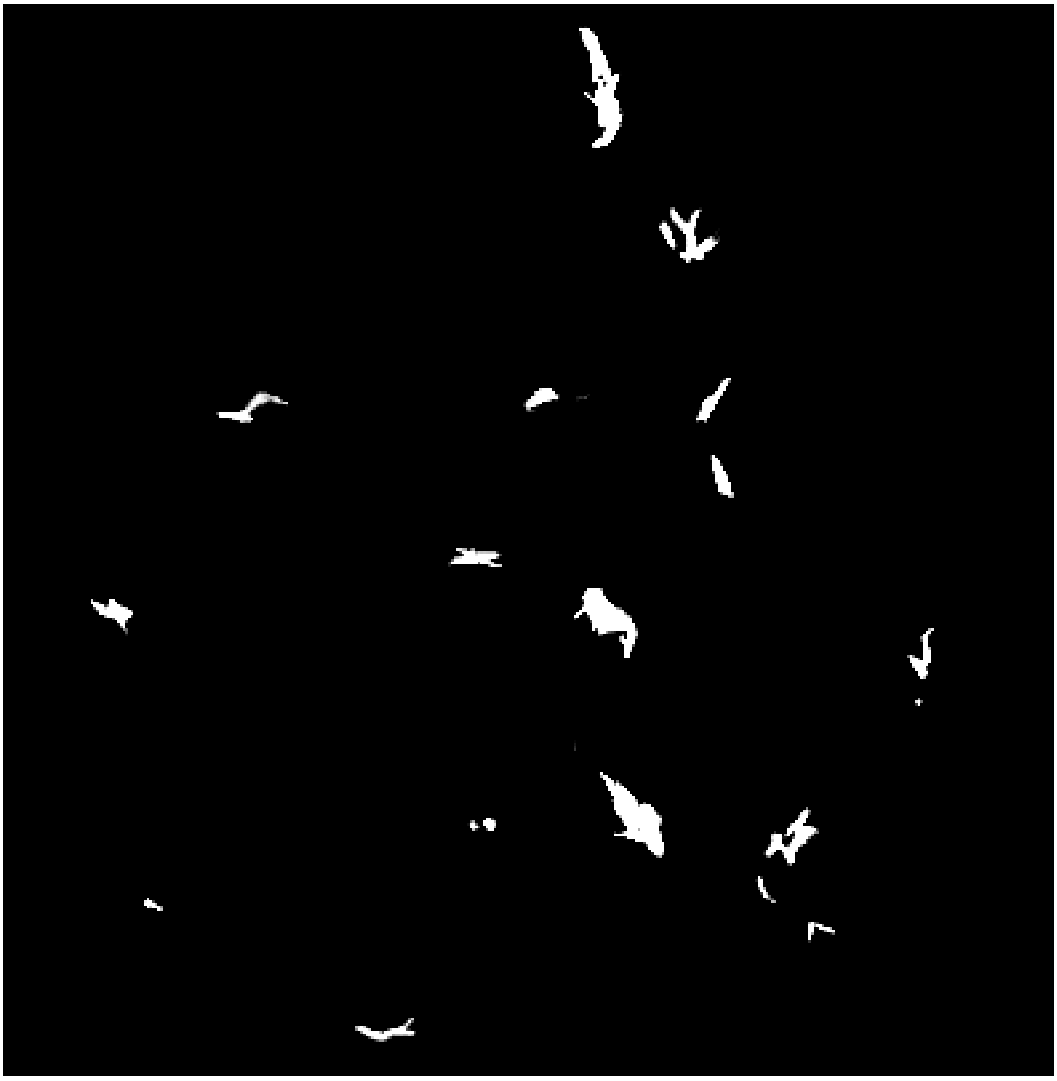

Figure S3. Omitting of SG dye-stained host RAW264.7 cell nuclei with empirically/visually determined PV of 150. See the original image of Fig. S1.

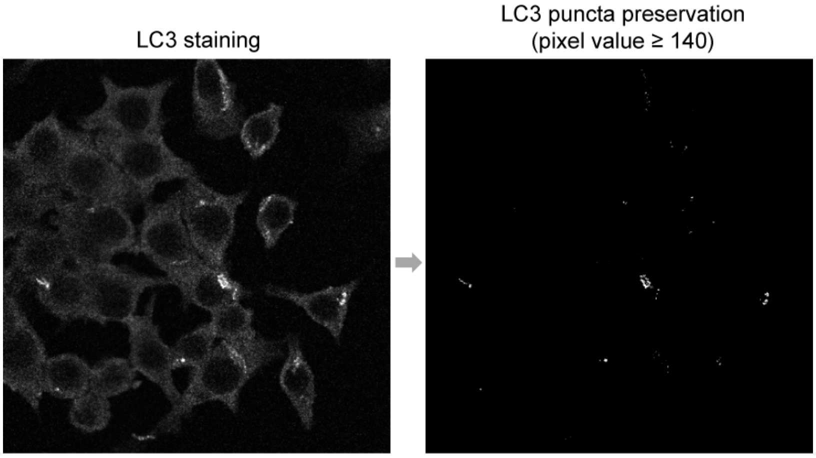

Figure S4. An empirically/visually-determined cutoff PV of LC3 could be used to omit background signal of LC3 and still preserve/represent LC3 puncta.

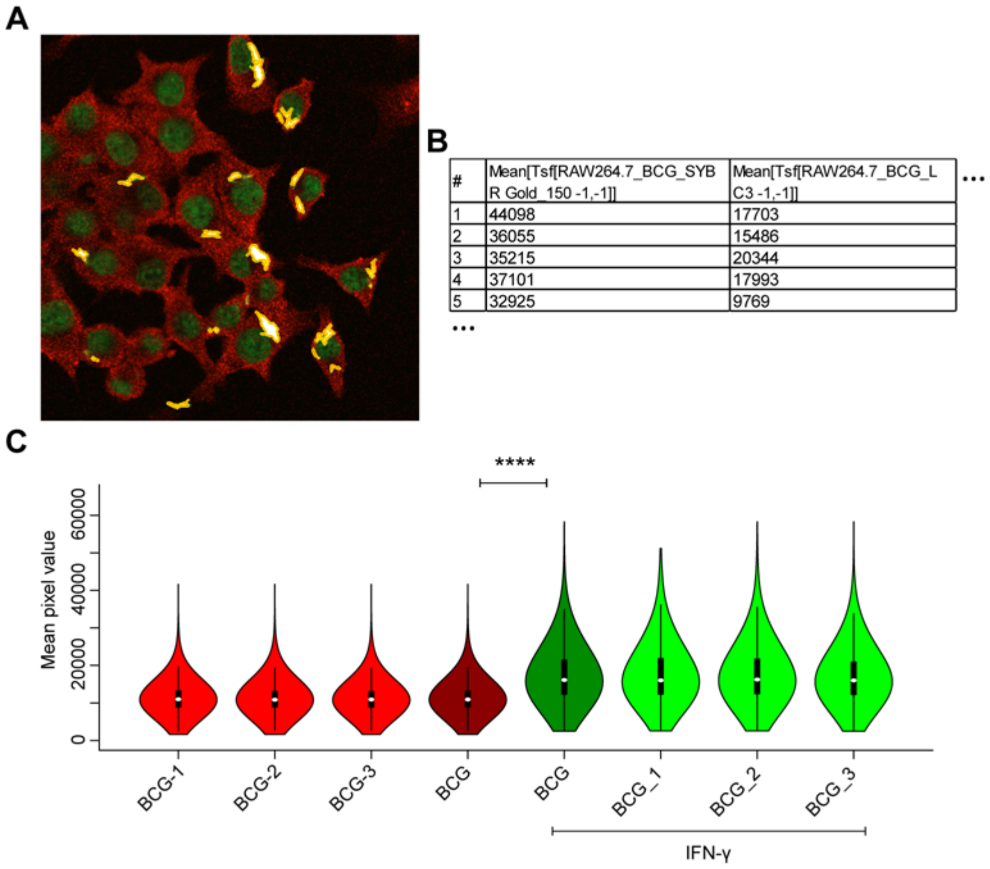

#	Mean[Tsf[RAW264.7_BCG_SYBR Gold_150 -1,-1]]	Mean[Tsf[RAW264.7_BCG_LC3 -1,-1]]
1	44098	17703
2	36055	15486
3	35215	20344
4	37101	17993
5	32925	9769

Figure S5. Co-localization analysis between intracellular BCG and LC3 using BioTek Gen5 Image Prime. (**A**) Locating the region of intracellular BCG/phagosomes with yellow margins. The representative figures used were Supplementary Figs. S1 - S3. (**B**) A partial table computing the cognate mean PVs of intracellular BCG (column 2) and LC3 (column 3) of panel A. (**C**) Violin plots of mean PVs of LC3 co-localized with intracellular BCG/phagosomes. This is the validation analysis of the pictures from the upper panel of Fig. 3a. In that experiment, RAW264.7 cells were infected with BCG and then either treated with IFN-γ or not. BCG_1~3, and BCG have the same meaning as in Fig. 3a. ****$P \leq 0.0001$; one-way ANOVA with Tukey's multiple comparisons test.

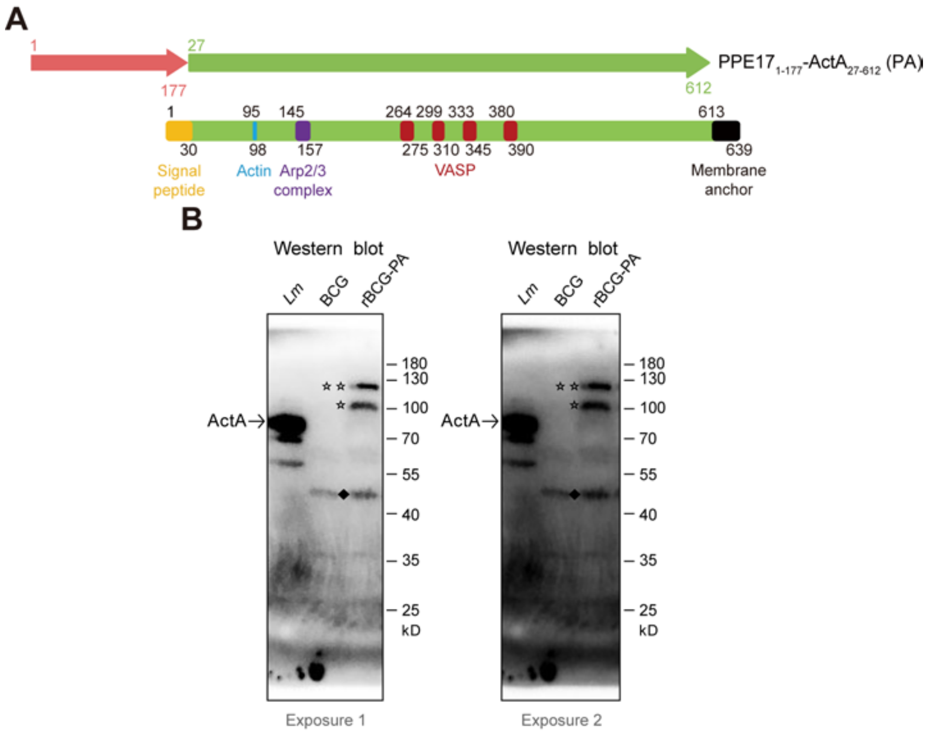

Figure S6. The construction of recombinant BCG strain rBCG-PA via the N-terminal domain of PPE17 ($PPE17_{1\text{-}177}$). **(A)** Schematic of $PPE17_{1\text{-}177}$-$ActA_{27\text{-}612}$ (PA). *Listeria monocytogenes* ActA (639 amino acids in length) with its domains involved in actin nucleation is schematically displayed at the bottom of panel A[4]. Arp2/3 complex = actin-related protein complex. VASP = vasodilator-stimulated phosphoprotein. (**B**) Validation of rBCG-PA via Western blotting. ◆ indicates non-specific blotting; ☆ and ☆☆: probably two translation products of *PA* with different post-translation modifications (the predicted molecular weight of PA is 83.3 kD).

```
public double[] customFunc(float[] c1, float[] c2) {
	/*c1 and c2 are arrays of pixel values of
	fluorescence channels in the same cell*/
	double maxVal = maximum_value(c2);
	double medianVal = median_value(c2);
	return new double[]{maxVal, medianVal};
}
```

Figure S7. Parts of the custom Java code for exporting maximum and median PVs of fluorescence channel 2. “c2” in bold could be changed to “c1” (for custom Java code of 2 or 3 reporter channels) or “c3” (for custom Java code of 3 reporter channels) for exporting maximum and median PVs of fluorescence channels 1 or 3 respectively.

Supplementary table

Table S1. Comparing the performance of "Custom Java code for 2 reporter channels" and "Custom Java code for 3 reporter channels".

Under "2 Reporter Channels"				
Label	Avg.Int.C1	Avg.Int.C2	Custom1	Custom2
image 1: cell 1	245.857	76.857	126	66
image 1: cell 2	252.557	85.889	211	78
image 1: cell 3	245.444	70.222	125	63.5
image 1: cell 4	246.588	96.294	218	83
image 1: cell 5	250.731	70.808	205	62.5
image 1: cell 6	251.172	99.828	213	91
image 1: cell 7	247.375	61.75	119	60
image 1: cell 8	243.855	67.754	167	61
image 1: cell 9	252.81	100.492	255	94
image 1: cell 10	250.942	98.231	242	85.5
image 1: cell 11	243.91	55.577	189	49.5
image 1: cell 12	252.413	87.492	208	83
image 1: cell 13	248.235	60.852	162	58
image 1: cell 14	253.465	130.564	255	120
image 1: cell 15	253.013	129.908	255	119.5
image 1: cell 16	250.188	126.826	255	111
image 1: cell 17	240.5	72.25	99	75
image 1: cell 18	253.261	73.359	207	67
image 1: cell 19	250.069	76.786	231	70
image 1: cell 20	250.722	174.167	255	179
Exported using "Custom Java code for 2 reporter channels".				
Ave.Int.C1: mean pixel value of fluorescence channel 1 (i.e. SYBR Gold).				
Ave.Int.C2: mean pixel value of fluorescence channel 2. (i.e. LC3).				
Custom1: maximum pixel value of fluorescence channel 2 (i.e. LC3).				
Custom2: median pixel value of fluorescence channel 2 (i.e. LC3)				

Under "3 Reporter channels"					
Label	Avg.Int.C1	Avg.Int.C2	Avg.Int.C3	Custom1	Custom2
image 1: cell 1	245.857	76.857	76.857	126	66
image 1: cell 2	252.557	85.889	85.889	211	78
image 1: cell 3	245.444	70.222	70.222	125	63.5
image 1: cell 4	246.588	96.294	96.294	218	83
image 1: cell 5	250.731	70.808	70.808	205	62.5
image 1: cell 6	251.172	99.828	99.828	213	91
image 1: cell 7	247.375	61.75	61.75	119	60
image 1: cell 8	243.855	67.754	67.754	167	61
image 1: cell 9	252.81	100.492	100.492	255	94
image 1: cell 10	250.942	98.231	98.231	242	85.5
image 1: cell 11	243.91	55.577	55.577	189	49.5
image 1: cell 12	252.413	87.492	87.492	208	83
image 1: cell 13	248.235	60.852	60.852	162	58
image 1: cell 14	253.465	130.564	130.564	255	120
image 1: cell 15	253.013	129.908	129.908	255	119.5
image 1: cell 16	250.188	126.826	126.826	255	111
image 1: cell 17	240.5	72.25	72.25	99	75
image 1: cell 18	253.261	73.359	73.359	207	67
image 1: cell 19	250.069	76.786	76.786	231	70
image 1: cell 20	250.722	174.167	174.167	255	179
Exported using "Custom Java code for 3 reporter channels", where "c2" was changed to "c3" (Supplementary Fig. S7).					
Ave.Int.C1: mean pixel value of fluorescence channel 1 (i.e. SYBR Gold).					
Ave.Int.C2: mean pixel value of fluorescence channel 2. (i.e. LC3).					
Ave.Int.C3: mean pixel value of fluorescence channel 3. (i.e. LC3).					
Custom1: maximum pixel value of fluorescence channel 3 (i.e. LC3).					
Custom2: median pixel value of fluorescence channel 3 (i.e. LC3)					

References

1. Fan, X. Y. *et al.* A novel differential expression system for gene modulation in Mycobacteria. *Plasmid* **61**, 39-46 (2009).

2. Ma, H. *et al.* Dose of incorporated immunodominant antigen in recombinant BCG impacts modestly on Th1 immune response and protective efficiency against Mycobacterium tuberculosis in mice. *J. Immunol. Res.* **2014**, 196124 (2014).

3. Hess, J. *et al.* Mycobacterium bovis Bacille Calmette-Guerin strains secreting listeriolysin of Listeria monocytogenes. *Proc. Natl. Acad. Sci. U. S. A.* **95**, 5299-5304 (1998).

4. Yoshikawa, Y. *et al.* Listeria monocytogenes ActA-mediated escape from autophagic recognition. *Nat. Cell Biol.* **11**, 1233-1240 (2009).
